# The relationship between physical functional capacity and lung function in obese children and adolescents

**DOI:** 10.1186/1471-2466-14-199

**Published:** 2014-12-15

**Authors:** Mariana Simões Ferreira, Roberto Teixeira Mendes, Fernando Augusto de Lima Marson, Mariana Porto Zambon, Ilma Aparecida Paschoal, Adyleia Aparecida Dalbo Contrera Toro, Silvana Dalge Severino, Maria Ângela Gonçalves de Oliveira Ribeiro, José Dirceu Ribeiro

**Affiliations:** Department of Pediatrics, Medical Sciences College, University of Campinas, Tessália Vieira de Camargo, 126, 13081-970 P.O. Box: 6111, Campinas, SP Brazil; Department of Genetics, Medical Sciences College, University of Campinas, Tessália Vieira de Camargo, 126, 13081-970 P.O. Box: 6111, Campinas, SP Brazil; Department of Medical Clinics, Medical Sciences College, University of Campinas, 13081-970 P.O. Box: 6111, Campinas, SP Brazil

**Keywords:** Childhood obesity, Lung function, Six-minute walk test

## Abstract

**Background:**

There is no consensus regarding obesity repercussions for lung function in children and adolescents. Therefore, the aim of the study was to determine whether obesity is associated with poor physical conditioning and damaged lung function in children and adolescents, and to correlate lung function with six-minute walk test (6MWT) results.

**Methods:**

This cross-sectional study included 38 obese subjects of both sexes, ranging between 5 and 17 years of age, as well as 56 control subjects paired by sex and age for the 6MWT, and 39 subjects for spirometry. Subjects performed spirometry according to the guidelines of the American Thoracic Society (ATS) and the European Respiratory Society**.** The obese group repeated spirometry after receiving bronchodilator (BD) treatments. Physical performance was evaluated via the 6MWT according to ATS guidelines.

**Results:**

The obese group demonstrated lower forced expiratory volumes in the first second compared with the control group based on forced vital capacity indices (p < 0.01), findings consistent with airway obstruction in 36.8% of patients in the obese group. Walking distances were shorter in the obese group than in the control group. Changes in lung function did not correlate directly with performance on the 6MWT among obese patients. However, there was a correlation between lung function and variables indicative of effort during exercise.

**Conclusion:**

In the present study, the obese group walked shorter distances and demonstrated lower values in some markers of lung function. However, there is no relationship between their physical conditions and these test results. Therefore, we cannot conclusively state that poor physical performance results from damaged pulmonary function.

**Electronic supplementary material:**

The online version of this article (doi:10.1186/1471-2466-14-199) contains supplementary material, which is available to authorized users.

## Background

Obesity is one of the biggest public health problems worldwide. It currently affects all age groups, including children and adolescents. The World Health Organization (WHO) characterizes the fight against obesity as one of the primary challenges for healthcare professionals in the 21st century. In Brazil, the prevalence of obesity is greater than 30% among children between 5 and 9 years of age and is almost 20% in children between 10 and 19 years of age [1–4].

Body mass is modulated from birth to adulthood by physiological mechanisms such as balancing intake, caloric expenditure and energy reserves. Hypercaloric diets and sedentary lifestyle have resulted in the development of obesity in younger populations. The development of obesity triggers a vicious cycle in which subjects become obese, and the systemic repercussions of their disease process make them intolerant to exercise; therefore, they become more sedentary, which promotes additional weight gain. Multisystem dysfunction, an entity previously observed only in adults, has become more common among children and adolescents, resulting in physical exercise intolerance and increasing the prevalence of obesity, which affects the cardiorespiratory system [2, 5].

In adults, obesity’s effects on lung function are well known. According to the Brazilian Pulmonary Function Guidelines, changes in spirometry occur only in the setting of morbid obesity, wherein low vital capacity (VC) and expiratory reserve volume (ERV) may be observed. Among children and adolescents, there is no literature consensus regarding common spirometric findings. Additionally, it is not clear when obesity begins to damage lung function, nor is it clear when patients’ physical performances become inadequate [6, 7].

Physical activity reduces the harm caused by obesity, which improves patients’ metabolic profiles and prevents obesity’s deleterious structural and psychosocial effects. Additionally, daily exercise improves quality of life [8].

In this study, lung function was assessed by spirometry, which measures inspired and expiratory air volumes and respiratory flow. It also helps in the prevention of ventilator disturbances, diagnosis and quantification [7]. The six-minute walk test (6MWT) was used to evaluate physical conditioning. The test demonstrates good reproducibility in children and adolescents; its application is inexpensive and simple and provides information about the global and integrated responses of each body system during exercise [9, 10].

Therefore, the principal aim of the present study was to evaluate obesity’s influence on physical conditioning and lung function in children and adolescents to determine if any correlation among these variables exists and to compare said values with those of a control group. Another point of investigation involved the relationship among height, weight and body mass index (BMI) with lung function variables, as well as the relationship among height, weight and BMI with 6MWT variables.

## Methods

### Study design and inclusion criteria

This was a cross-sectional study that included 38 obese children and adolescents of both sexes between 5 and 17 years of age, as well as a control group paired by sex and age. Obese subjects were followed at the Multidisciplinary Ambulatory Service for Obese Children and Adolescents at University Hospital.

The CDC (Center for Disease Control and Prevention) standards for individuals between 2 and 19 years of age were used to define obesity: a subject is considered obese if his BMI is above the 95th percentile.

The control group was composed of healthy subjects of the same age groups. Control individuals had previously registered with the Pulmonary Physiology Laboratory database. The spirometry (n = 39) and 6MWT (n = 56) control groups consisted of distinct individuals, so they cannot be considered a single group.

### Exclusion criteria

Patients with acute or chronic diseases, neurological or physical limitations, or any respiratory diseases that may have interfered with their ability to perform spirometry or to complete the 6MWT were excluded from this study.

### Procedures

The study was approved by the Ethics Committee of the Medical Sciences College of Unicamp (#1165/2009). The parents and guardians of each of the participants provided written informed consent prior to the evaluations.

Each subject performed spirometry to assess pulmonary function. They were subsequently scheduled to complete a 6MWT on another date in order to prevent the use of bronchodilators (BDs), spirometry or fatigue from interfering with their performances during the walking test. Of the 38 patients who underwent spirometry, 29 completed the 6MWT.

### Spirometry

Spirometry was performed using the CPFS/D model spirometer (MedGraphics, Saint Paul, Minnesota, USA, software BREEZE PF 3.8 B version for Windows 95/98/NT), and the results were assessed using the American Thoracic Society (ATS) and European Respiratory Society (ERS) standards.

Subjects who performed spirometry were allowed to rest for 10 min before beginning testing. The mouthpiece was properly positioned on the tongue, and it was ensured that the lips were tightly sealed around the mouthpiece to avoid air leakage. During the evaluation, subjects remained standing and performed slow and forced maneuvers. Following the first measurement, the obese group underwent salbutamol inhalation and repeated the test after 20 min. Acceptance criteria included the generation of at least three acceptable and two reproducible curves. Percentages of predicted values were used for statistical analysis.

The variables analyzed included the following: forced vital capacity (FVC), forced expiratory volume in the first second (FEV_1_), FEV_1_/FVC index, forced expiratory flow at 25%, 50% and 75% of FVC (FEF_25%_, FEF_50%_, FEF_75%_), forced expiratory flow between 25% and 75% of FVC (FEF_25–75%_), maximal forced expiratory flow (FEF_max_), and expiratory reserve volume (ERV).

### The 6 minute walk test (6MWT)

The 6MWT was performed according to ATS guidelines. Walking distance (WD), work index (W = body weight X WD), respiratory rate (RR), heart rate (HR), saturation of peripheral oxygen (SpO_2_), physiological cost index (PC = HR_6minutes -_ HR_rest_/average speed) and dyspnea perception (BorgD) based on the Borg scale were evaluated. Arterial blood pressure (BP) and legs effort perception (BorgL) were also recorded in the obese group. Measurements were performed at rest, immediately following the 6MWT and following three min of rest.

### Statistical analysis

Our statistical analysis compared height, weight and BMI with lung function variables (FVC, FEV_1_, FEV_1_/FVC index, FEF_25%_, FEF_50%_, FEF_75%_, FEF_25–75%_, FEF_max_ and ERV): height, weight and BMI were also compared with 6MWT variables (WD, W, RR, HR, SpO_2_, PC, BorgD, BP and BorgL). Correlation tests were performed using the following variables: (i) height, weight, BMI and lung function; (ii) height, weight, BMI and 6MWT; and (iii) lung function and 6MWT.

SPSS 21.0 (SPSS Inc., Chicago, IL, EUA) was used to tabulate data. The Mann–Whitney test was used to compare two numeric variables, and the Kruskal-Wallis test evaluated three or more groups. The χ^2^ test was used for categorical variables analysis. The Spearman correlation was used to determine relationships between variables. The Wilcoxon test compared pulmonary function before and after BD use. α (alpha) was equal to 0.05, and the Bonferroni correction was used for multiple tests.

The non-parametric test was performed to determine sample distribution. The data demonstrated a non-parametric distribution following an analysis using the Kolmogorov-Smirnov test of normality and the Shapiro-Wilk test of normality, which accounted for the graphic analysis for the distribution of data.

The sample size was calculated using G*Power 3.1.9.2. After taking into account the number of subjects enrolled (38 obese and 56 healthy controls) for all tests performed, the power was greater than 0.80. For the Mann–Whitney test, given an α = 0.05 and an effect size, d = 0.80, a power, β, equal to 0.96 was achieved. For the Kruskal-Wallis test, given an α = 0.05, a number of groups = 4, an effect size, f = 0.40, a power, β, equal to 0.90 was achieved. For the Wilcoxon test, given an α = 0.05, an effect size, dz = 0.50, a power, β, equal to 0.99 was achieved. For the Spearman Regression, given an α = 0.05, a correlation, ρH1 = 0.3, and a correlation, ρH0 = 0, a power, β, equal 0.84 was achieved.

## Results

The results demonstrate relationships with positive p values in the tables considering both the obese group and the control group. In cases of relationships noted with positive p values that were grouped by both sex and age, the data are shown within the figures. All significant and non significant p values are described for each of the analyzed variables.

### Sample description

**Figure 1:**
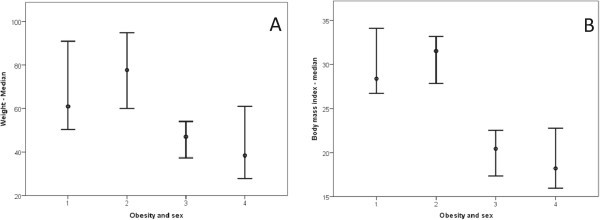
**Obesity based on sex.** The follow clinical markers were analyzed: weight and body mass index. In all cases, as follows: 1 = female/obese (18 patients); 2 = male/obese (20 patients); 3 = female/eutrophic (21 patients); 4 = male/eutrophic (18 patients). **(A)** Weight = 1 ≠ 3,4; 2 ≠ 3,4; 3 ≠ 1,2; 4 ≠ 1,2. Median: (1) 60.95; (2) 77.75; (3) 48.00; (4) 37.70. **(B)** Body mass index = 1 ≠ 3,4; 2 ≠ 3,4; 3 ≠ 1,2; 4 ≠ 1,2. Median: (1) 28.36; (2) 31.51; (3) 20.69; (4) 18.15.

**Figure 2:**
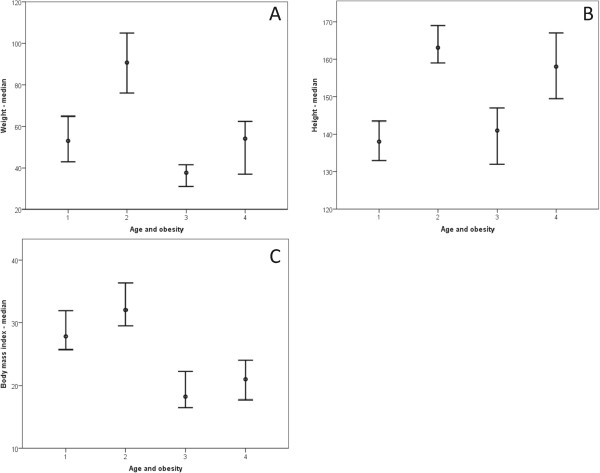
**Obesity based on patients’ ages.** The following clinical markers were analyzed: weight, height and body mass index. In all cases, as follows: 1 = age between five and 11 years old/obese (17 patients); 2 = age between 11 and 17 years old/obese (21 patients); 3 = age between five and 11 years old/eutrophic (20 patients); 4 = age between 11 and 17 years old/eutrophic (19 patients). **(A)** Weight = 1 ≠ 2,3; 2 ≠ 1,3,4; 3 ≠ 1,2,4; 4 ≠ 2,3. Median: (1) 53; (2) 90.60; (3) 37.40; (4) 54.10. **(B)** Height = 1 ≠ 2,4; 2 ≠ 1,3; 3 ≠ 2,4; 4 ≠ 1,3. Median: (1) 138; (2) 163; (3) 143; (4) 157.5. **(C)** Body mass index = 1 ≠ 2,3,4; 2 ≠ 1,3,4; 3 ≠ 1,2; 4 ≠ 1,2. Median: (1) 27.83; (2) 32.05; (3) 18.21; (4) 21.05.

The study included 38 [20 (52.13%) male] obese children and adolescents ranging from five to 17 years of age. All subjects performed spirometry, and 29 completed the 6MWT, a sample loss of 23.48% (nine patients). Table 1 presents patient and control subject characterizations relative to spirometry data, and Table 2 describes the 6MWT data. Figure 1 presents body weight (Figure 1A) and BMI (Figure 1B) relationships based on sex, and Figure 2 presents body weight (Figure 2A), height (Figure 2B), and BMI (Figure 2C) relationships based on age groups.

**Table 1:** Spirometry* characterization groups according to sex and age

	Obese		Eutrophic	
	Male	Female	Male	Female
N	20	18	18	21
FVC	104.50	107.50	99.50	97
FEV_1_	95	101	103.50	106
FEV_1_/FVC	86.50	92	97	99
FEF_25%_	76	99	102	111
FEF_50%_	68.50	90	98.50	105
FEF_75%_	53.50	78	84	96
FEF_25–75%_	73.50	95	109.50	122
FEF_max_	83	103.50	108.50	112
VER	111	133.50	92	157
	5 to 11 years old	11 to 17 years old	5 to 11 years old	11 to 17 years old
N	17	21	20	19
FVC	100	109	97	101
FEV_1_	97	101	102.50	107
FEV_1_/FVC	90	86	101	97
FEF_25%_	88	78	117.50	106
FEF_50%_	84	74	105.50	98
FEF_75%_	68	57	93.50	88
FEF_25–75%_	78	84	118.50	116
FEF_max_	97	90	119	106
VER	104	161	99	138

N = number of patients; FVC = forced vital capacity; FEV_1_ = forced expiratory volume at first second; FEF = forced expiratory flow; max = maximum; ERV = expiratory reserve volume. *Spirometry values are shown as percentages of predicted values.

**Table 2:** **6MWT characterization groups according to sex and age**

	Obese		Eutrophic	
	Male	Female	Male	Female
N	16	13	25	31
WD	520.56	528.57	690	665
W	41,867.54	30,014.33	30,222	24,735
PC	0.36	0.35	0.35	0.37
HR - rest	83.5	90	94	90
RR - rest	18	17	18	20
SpO_2_ - rest	98	98	98	98
Borg - rest	0	0	0	0
HR - 6'	118	123	129	136
RR - 6'	24	23	16	30
SpO2 - 6'	98	98	98	98
Borg - 6'	2	3	3	3
HR - 9'	89	94	96	102
RR - 9'	18	17	20	22
SpO2 - 9'	98	98	98	98
Borg - 9'	0	0	0.50	1
	5 to 11 years old	11 to 17 years old	5 to 11 years old	11 to 17 years old
N	13	16	27	29
WD	515.15	529.21	665	675
W	29,044.85	46,741.59	20,160	31,500
PC	0.21	0.39	0.42	0.32
HR - rest	96	82	88	96
RR - rest	20	16	20	20
SpO_2_ - rest	98	98	98	98
Borg - rest	0	0	0	0
HR - 6'	118	120	135	130
RR - 6'	28	23	28	28
SpO_2_ - 6'	98	98	98	98
Borg - 6'	3	2	3	3
HR - 9'	96	88	100	98
RR - 9'	19	17	21	20
SpO2 - 9'	98	98	98	98
Borg - 9'	0	0	1	0.50

N = number of patients; WD = walking distance; W = work index; PC = physiological cost; HR = heart rate; RR = respiratory rate; SpO_2_ = oxygen saturation; Borg = dyspnea perception according to Borg scale; 6’ = six minutes; 9’ = nine minutes.

### Spirometry

**Figure 3:**
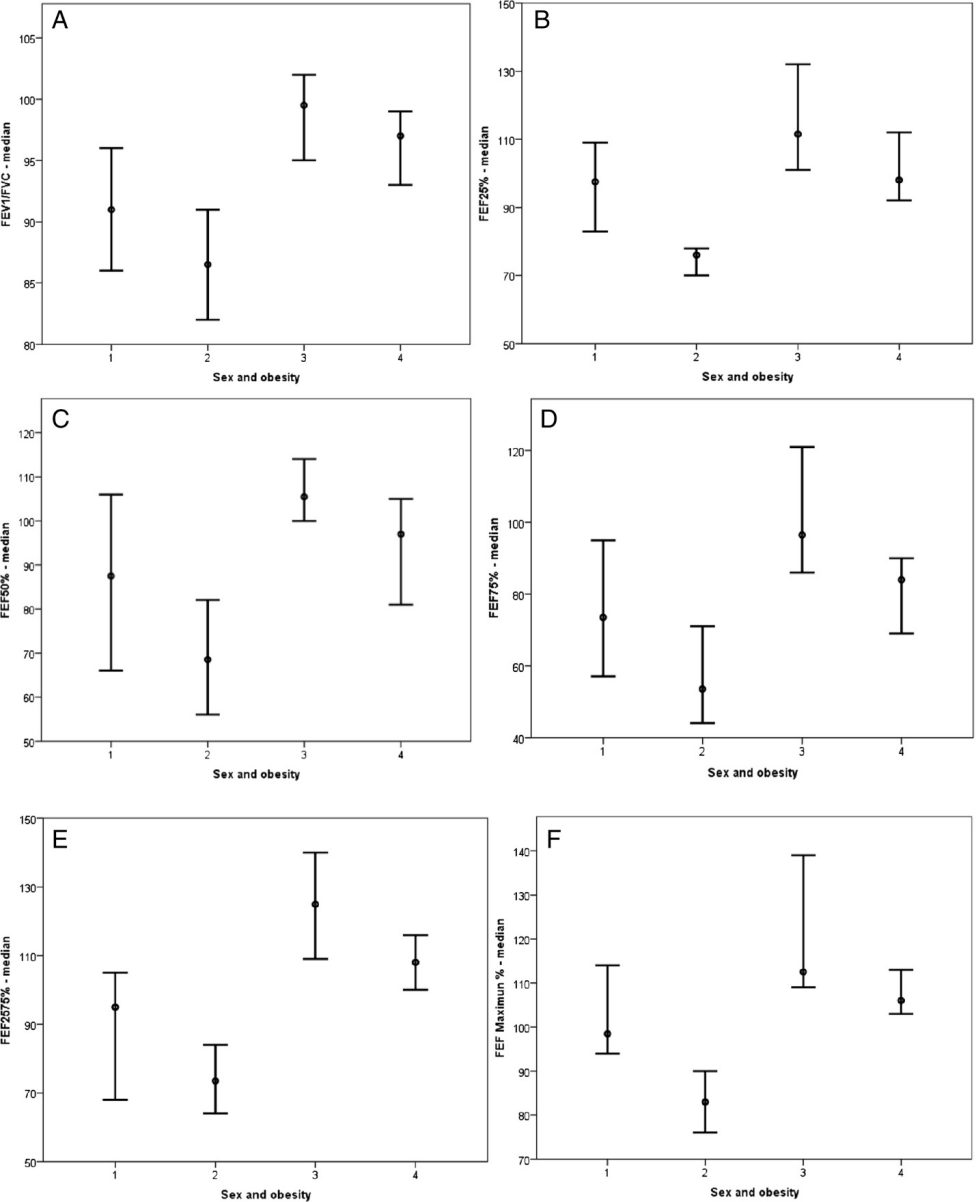
**Obesity based on sex.** The clinical markers analyzed included spirometry parameters. In all cases, as follows: 1 = female/obese (18 patients); 2 = male/obese (20 patients); 3 = female/eutrophic (21 patients); 4 = male/eutrophic (18 patients). **(A)** FEV_1_/FVC = 1 ≠ 2,3,4; 2 ≠ 1,3,4; 3 ≠ 1,2; 4 ≠ 1,2. Median: (1) 91; (2) 86.50; (3) 100; (4) 97. **(B)** FEF_25_% = 1 ≠ 2,3,4; 2 ≠ 1,3,4; 3 ≠ 1,2,4; 4 ≠ 1,2,3. Median: (1) 97.50; (2) 76; (3) 111; (4) 102. **(C)** FEF_50_% = 1 ≠ 2,3; 2 ≠ 1,3,4; 3 ≠ 1,2; 4 ≠ 2. Median: (1) 87.50; (2) 68.50; (3) 105; (4) 94. **(D)** FEF_75_% = 1 ≠ 2,3; 2 ≠ 1,3,4; 3 ≠ 1,2,4; 4 ≠ 2,3. Median: (1) 73.50; (2) 53.50; (3) 96; (4) 82. **(E)** FEF_25–75_% = 1 ≠ 2,3,4; 2 ≠ 1,3,4; 3 ≠ 1,2,4; 4 ≠ 1,2,3. Median: (1) 95; (2) 73.50; (3) 122; (4) 107.50. **(F)** FEF maximum = 1 ≠ 2,3; 2 ≠ 1,3,4; 3 ≠ 1,2; 4 ≠ 2. Median: (1) 98.50; (2) 83; (3) 103; (4) 108.50.

**Figure 4:**
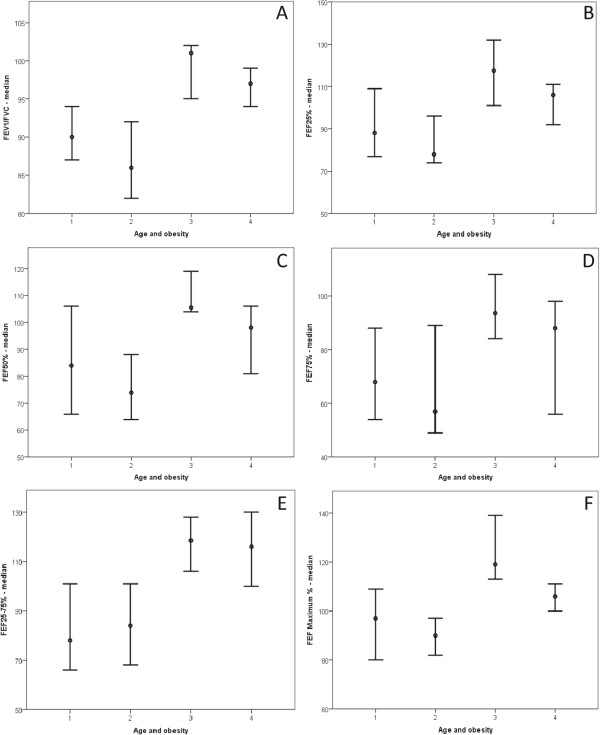
**Obesity based on patients’ ages.** The clinical markers analyzed included spirometry parameters. In all cases, as follows: 1 = age between five and 11 years old/obese (17 patients); 2 = age between 11 and 17 years old/obese (21 patients); 3 = age between five and 11 years old/eutrophic (20 patients); 4 = age between 11 and 17 years old/eutrophic (19 patients). **(A)** FEV_1_/FVC = 1 ≠ 3,4; 2 ≠ 3,4; 3 ≠ 1,2; 4 ≠ 1,2. Median: (1) 90; (2) 86; (3) 101; (4) 97. **(B)** FEF_25_% = 1 ≠ 3; 2 ≠ 3,4; 3 ≠ 1,2; 4 ≠ 2. Median: (1) 88; (2) 78; (3) 117; (4) 106. **(C)** FEF_50_% = 1 ≠ 3; 2 ≠ 3,4; 3 ≠ 1,2; 4 ≠ 2. Median: (1) 84; (2) 74; (3) 105; (4) 98. **(D)** FEF_75_% = 1 ≠ 3; 2 ≠ 3,4; 3 ≠ 1,2; 4 ≠ 2. Median: (1) 68; (2) 57; (3) 94; (4) 88. **(E)** FEF_25–75_% = 1 ≠ 3,4; 2 ≠ 3,4; 3 ≠ 1,2; 4 ≠ 1,2. Median: (1) 78; (2) 84; (3) 116; (4) 116. **(F)** FEF maximum = 1 ≠ 3; 2 ≠ 3,4; 3 ≠ 1,2,4; 4 ≠ 2,3. Median: (1) 88; (2) 78; (3) 117; (4) 106.

Spirometric variables were analyzed in both the obese and healthy groups (Table 3) and subdivided by sex (Figure 3) and age (Figure 4). Figure 3 describes spirometric variable relationships based on sex as follows: FEV_1_/FVC (Figure 3A); FEF_25%_ (Figure 3B); FEF_50%_ (Figure 3C); FEF_75%_ (Figure 3D); FEF_25–75%_ (Figure 3E); and FEFmax (Figure 3F). Relationships involving spirometric variables based on age are described in Figure 4 as follows: FEV_1_/FVC (Figure 4A); FEF_25%_ (Figure 4B); FEF_50%_ (Figure 4C); FEF_75%_ (Figure 4D); FEF_25–75%_ (Figure 4E); and FEF_max_ (Figure 4F).

**Figure 5:**
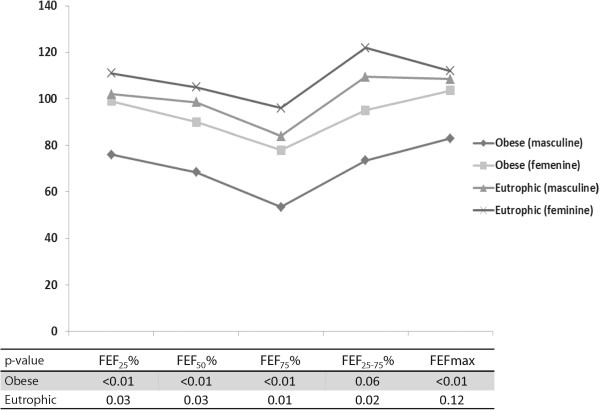
**Comparisons of median percentages of predicted forced expiratory flow values according to sex, followed by p-values.**

The evaluation of each group relative to subjects’ sexes (obese male versus obese female/healthy male versus healthy female) identified lower values of forced expiratory flow in male subjects compared with female subjects in both groups (Figure 5). There were no differences in ERV between obese and healthy subjects.

**Table 3:** **Relationship between obese and eutrophic subjects regarding weight, body mass index, and spirometry parameters with a positive p-value**

Clinical variables	Groups	N	Mean	Standard deviation	Median	CI		Minimum	Maximum	p-value
						5%	95%			
Weight	Obese	38	75.85	28.75	71.8	66.40	85.30	37	175	**<0.003**
	Eutrophic	39	44.513	13.96	40.7	39.99	49.04	18.60	69.30	
Body mass index	Obese	38	31.88	8.01	30.2	29.24	34.51	21.22	65.87	**<0.003**
	Eutrophic	39	19.91	3.76	18.15	18.68	21.12	14.19	22.79	
FEV_1_/FVC	Obese	38	89	7.17	88.5	86.64	91.36	74	105	**<0.003**
	Eutrophic	39	97.51	5.20	98	95.83	99.20	84	107	
FEF_25%_	Obese	38	87.06	21.42	80	80.01	94.09	40	154	**<0.003**
	Eutrophic	39	110.31	19.18	110	104.09	116.52	69	149	
FEF_50%_	Obese	38	80.53	25.41	77	72.17	88.88	32	150	**<0.003**
	Eutrophic	39	101.36	18.59	104	95.33	107.38	64	141	
FEF_75%_	Obese	38	69.42	28.53	64.5	60.04	78.80	19	138	**0.003**
	Eutrophic	39	90.97	28.16	90	81.85	100.10	42	156	
FEF_25–75%_	Obese	38	84.42	25.26	79	76.12	92.72	29	147	**<0.003**
	Eutrophic	39	116.28	22.85	116	108.88	123.69	72	159	
FEF maximum	Obese	38	94.37	19.94	92.5	87.81	100.92	56	162	**<0.003**
	Eutrophic	39	115.23	18.17	112	109.34	121.12	80	163	

CI = confidential interval; n = number of patients; FEV_1_ = forced expiratory volume in 1 second; FVC = forced vital capacity; FEF = forced expiratory flow. Statistical analyses were performed using the Mann–Whitney test, given an α = 0.05.

Regarding BD responses in obese subjects, decreased FVCs (p = 0.05) were observed, and the FEV_1_/FVC ratios (p = 0.01) were consequently increased. No improvements greater than 10% were observed for FEV_1_, findings suggesting that inhaled medication did not elicit improvements in lung function (Additional file 1). Additional file 2 presents each p value from the spirometric evaluations, and Additional file 3 depicts the relationships between BD pre and post values based on subjects’ sexes and ages.

### The Six minute walk test (6MWT)

**Figure 6:**
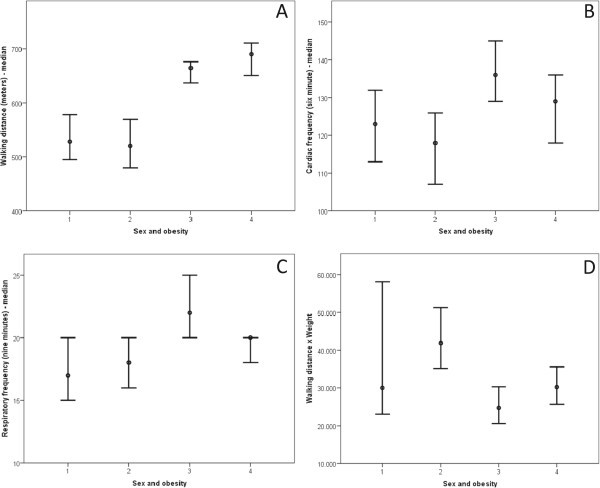
**Obesity based on sex. Clinical markers analyzed include walking distance, cardiac rate, respiratory rate and walking distance x weight.** In all cases, as follows: 1 = female/obese (13 patients); 2 = male/obese (16 patients); 3 = female/eutrophic (31 patients); 4 = male/eutrophic (25 patients). **(A)** Walking distance = 1 ≠ 3,4; 2 ≠ 3,4; 3 ≠ 1,2,4; 4 ≠ 1,2,3. Median: (1) 528.27; (2) 520.55; (3) 665; (4) 690. **(B)** Cardiac rate in six min = 1 ≠ 3; 2 ≠ 3,4; 3 ≠ 1,2; 4 ≠ 2. Median: (1) 123; (2) 118; (3) 136; (4) 129. **(C)** Respiratory rate in nine min = 1 ≠ 3; 2 ≠ 3; 3 ≠ 1,2,4; 4 ≠ 3. Median: (1) 17; (2) 18; (3) 22; (4) 20. **(D)** Walking distance x weight = 1 ≠ 2,3; 2 ≠ 1,3,4; 3 ≠ 1,2,4; 4 ≠ 2,3. Median: (1) 30,014.33; (2) 41,867.54; (3) 24,735; (4) 30.222.

**Figure 7:**
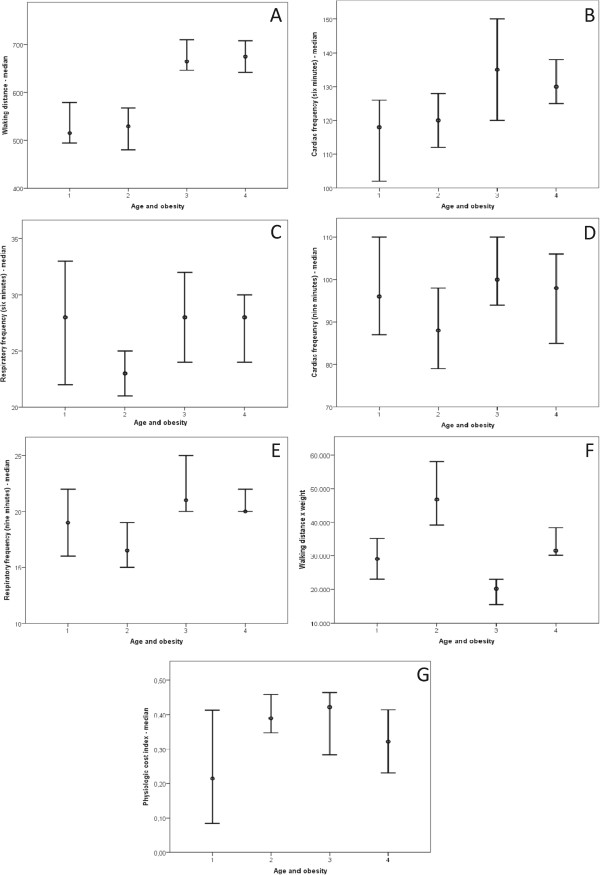
**Obesity based on patients’ ages.** Clinical markers analyzed include walking distance, cardiac rate, respiratory rate, walking distance x weight and physiology cost index. In all cases, as follows: 1 = age between five and 11 years old/obese (13 patients); 2 = age between 11 and 17 years old/obese (16 patients); 3 = age between five and 11 years old/eutrophic (27 patients); 4 = age between 11 and 17 years old/eutrophic (29 patients). **(A)** Walking distance = 1 ≠ 3,4; 2 ≠ 3,4; 3 ≠ 1,2; 4 ≠ 1,2. Median: (1) 515.15; (2) 529.21; (3) 665; (4) 675. **(B)** Cardiac rate in six min = 1 ≠ 3,4; 2 ≠ 3,4; 3 ≠ 1,2; 4 ≠ 1,2. Median: (1) 118; (2) 120; (3) 135; (4) 130. **(C)** Respiratory rate in six min = 1 ≠ 2; 2 ≠ 1,3,4; 3 ≠ 2; 4 ≠ 2. Median: (1) 28; (2) 23; (3) 28; (4) 28. **(D)** Cardiac rate in nine min = 2 ≠ 3,4; 3 ≠ 2; 4 ≠ 2. Median: (1) 96; (2) 88; (3) 100; (4) 98. **(E)** Respiratory rate in nine min = 1 ≠ 3; 2 ≠ 3,4; 3 ≠ 1,2; 4 ≠ 2. Median: (1) 19; (2) 16.5; (3) 21; (4) 20. **(F)** Walking distance x weight = 1 ≠ 2,3; 2 ≠ 1,3,4; 3 ≠ 1,2,4; 4 ≠ 2,3. Median: (1) 29,044.85; (2) 46,741.59; (3) 20,160; (4) 31,500. **(G)** Physiology cost index = 1 ≠ 2,3; 2 ≠ 1,4; 3 ≠ 1,4; 4 ≠ 2,3. Median: (1) 0.21; (2) 0.38; (3) 0.42; (4) 0.32.

The obese group walked a shorter distance than the healthy group during the 6MWT (p < 0.01) in spite of doing more work (p < 0.01) (Table 4). Differences were maintained after subdividing the groups by sex (Figure 6) and age (Figure 7). The 6MWT variable relationships based on sex are described in Figure 6 as follows: WD (Figure 6A), HR (six min) (Figure 6B), RR (nine min) (Figure 6C), and W (Figure 6D). Figure 7 describes the 6MWT variable relationships by age group as follows: WD (Figure 7A); HR (six min) (Figure 7B); HR (nine min) (Figure 7C); RR (six min) (Figure 7D); RR (nine min) (Figure 7E); W (Figure 7F); and PC (Figure 7G).

**Table 4:** **Relationship between obese and eutrophic subjects regarding weight, body mass index and spirometry parameters with a positive p-value**

Variables	Groups	N	Mean	Standard deviation	Median	CI		Minimum	Maximum	p-value
						5%	95%			
Walking distance	Obese	29	523.06	54.21	528.27	502.43	543.68	352.38	628.73	**<0.003**
	Eutrophic	56	669.66	45.68	669.00	657.43	681.89	565.60	771	
Cardiac frequency - 6´	Obese	29	119.07	14.63	118	113.50	124.64	79.00	147	**0.003**
	Eutrophic	56	133.48	19.45	132	128.27	138.69	88.00	173	
Respiratory frequency - 6´	Obese	29	25.38	5.50	23	23.29	27.47	16.00	40	**0.036**
	Eutrophic	56	28.68	6.79	28	26.86	30.50	18.00	58	
Cardiac frequency - 9´	Obese	29	92.14	10.96	93	87.97	96.31	75.00	112	**0.042**
	Eutrophic	56	99.79	13.61	99	96.14	103.43	76.00	127	
Respiratory frequency - 9´	Obese	29	18.00	3.07	18	16.83	19.17	10.00	24	**<0.003**
	Eutrophic	56	21.37	4.69	20	20.12	22.63	12.00	32	
Work	Obese	29	40,630.81	14,853.16	37,678.27	34,980.97	46,280.65	18,998.78	84,192	**<0.003**
	Eutrophic	56	28,069.87	9,885.54	28,233.75	25,421.81	30,716.54	13,724.10	55,335	

CI = confidential interval; n = number of patients. Statistical analyses were performed using the Mann–Whitney test, given an α = 0.05.

There were no differences in PC between the groups. However, subdividing them by age revealed higher PC values among subjects between five and 11 years old (p = 0.01). However, PC values were much higher among obese subjects between 11 and 17 years of age (p = 0.02).

**Figure 8:**
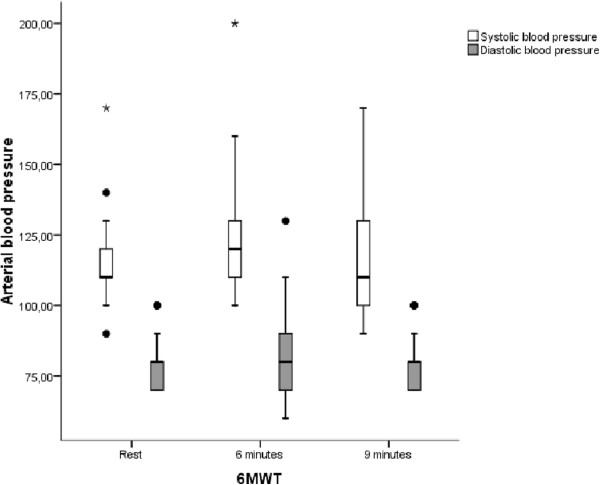
**Comparisons of blood pressure variations in the obese group, analyzing three different moments of the 6MWT performance.**

**Figure 9:**
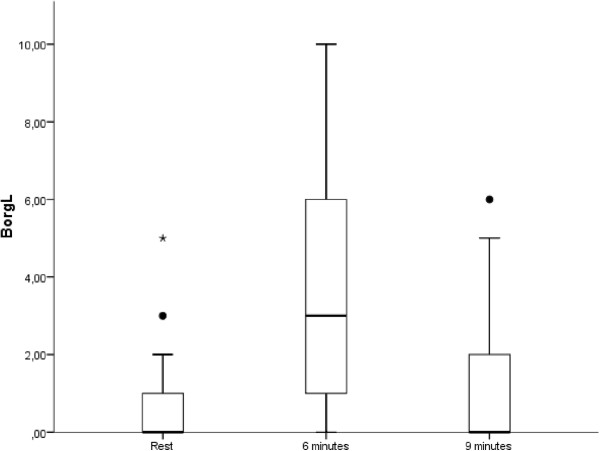
**Comparisons of the legs effort perception in the obese group according to the Borg scale, analyzing three different moments of the 6MWT performance.**

Systolic and diastolic BP and BorgL were evaluated in the obese group. The behaviors of the following variables over time are included in Figure 8 (systolic and diastolic BP) and Figure 9 (BorgL).

Additional file 4 shows all 6MWT variables’ p values in both groups, while Additional file 5 shows 6MWT test markers p values only in obese group.

**Figure 10:**
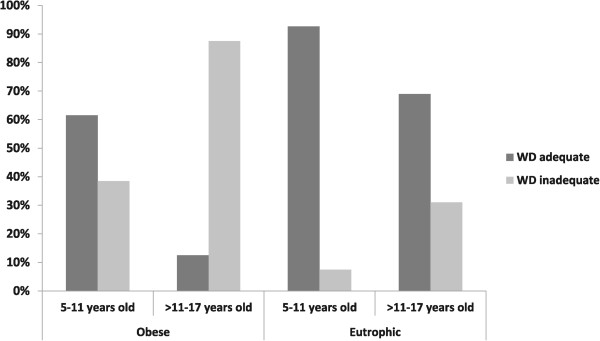
**Comparisons of obese and eutrophic 6MWT performances based on age group using the criteria developed by Priesnitz** ***et al.***

There is no consensus in the literature regarding normal 6MWT standards for children or adolescents. Therefore, the performances of our study subjects were analyzed based on four different normality equations (Table 5), equations that reflected disagreement and demonstrated the impracticality of their being used for this analysis. In order to compare the patients of the obese group, the equation developed by Priesnitz et al. [11] was utilized, as their study population was similar to that of the present study and included body weight as a subtractive variable (Figure 10) [11–14]. Table 6 demonstrates the differences among the normality equations based on the variables utilized and the age groups under consideration.

**Table 5:** **Percentage of subjects with inadequate performance according to four normality equations and odds-ratios**

Li et al. (2007)			Geiger et al. (2007)			Priesnitz et al. (2009)			Oliveira (2007)		
Obese	Eutrophic	OR (5-95% CI)	Obese	Eutrophic	OR (5-95% CI)	Obese	Eutrophic	OR (5-95% CI)	Obese	Eutrophic	OR (5-95% CI)
100%	83.9%	-	93.1%	35.7%	10.04 (4.54-24.95)	65.5%	19.6%	7.34 (3.91-14.17)	82.8%	42.9%	6.41 (3.36-12.6)
**p = 0.02**			**p < 0.01**			**p < 0.01**			**p < 0.01**		

OD = odds-ratio; CI = confidential interval.

**Table 6:** **Comparisons among normality equations, study age groups, nationalities and normality equation variables**

Authors	Publication year	Nationality	Age group	Variables
Li et al.	2007	Chinese	7-16 years old	Sex, ∆HR and height.
Geiger et al.	2007	Austrian	3-18 years old	Age and height.
Priesnitz et al.	2009	Brazilian	6-12 years old	Age, height, ∆HR and body weight.
Oliveira	2007	Brazilian	7-17 years old	Sex, age and BMI.

∆HR = pre and post walking heart rate difference; BMI = body mass index.

Significant correlations between lung function and physical performance are demonstrated in Table 7. There were no significant correlations between subjects’ performances (WD) and lung function variables. We observed a relationship only between lung function and variables indicative of effort.

**Table 7:** **Correlation between spirometric variables and 6MWT variables**

	FVC	FEV _1_	FEV _1_/FVC	FEF _25%_	FEF _50%_	FEF _75%_	FEF _max_	ERV
WD	0.187	0.140	0	0.231	0.119	0.029	0.231	**0.375**
W	0.214	−0.125	−0.381	**−0.384**	−0.297	−0.300	**−0.398**	0.357
Rest								
Systolic BP	0.118	−0.239	−0.317	−0.284	−0.260	**−0.361**	−0.251	0.029
Diastolic BP	**0.544**	0.124	−0.313	−0.037	−0.307	−0.199	−0.035	−0.002
HR	−0.016	−0.182	−0.200	0.050	−0.155	−0.334	−0.048	**−0.432**
Borg	0.061	**−0.366**	**−0.536**	−0.168	**−0.415**	−0.512	−0.096	0.089
6’ minutes								
Systolic BP	0.135	−0.261	**−0.376**	**−0.366**	**−0.423**	−0.357	−0.251	−0.134
9’ minutes								
HR	−0.008	−0.052	−0.041	0.257	−0.013	−0.178	0.054	**−0.367**
BorgL	**0.433**	0.239	−0.131	−0.166	−0.065	0.057	−0.085	0.154

FVC = forced vital capacity; FEV_1_ = forced expiratory volume at first second; FEF = forced expiratory flow; % = percentage; max = maximum; ERV = expiratory reserve volume; WD = walking distance; W = work; BP = blood pressure; HR = heart rate; Borg = dyspnea perception according to Borg scale; 6’ = six minutes; 9’ = nine minutes; BorgL = legs effort perception according to Borg scale. The Spearman correlation was used to determine relationships among variables. Positive p-values are shown in bold.

## Discussion

Physiological pulmonary capacity is dependent on body size and system efficiency. Therefore, exercise adaptation is influenced by body growth and pubertal development. Obesity is related to sedentary lifestyle, which effects performance, and also to increased fat mass relative to muscle mass per unit of weight. Being overweight makes any physical activity uncomfortable and reduces physical activity interest, which fosters the development of a vicious cycle [15].

Maximal oxygen uptake (VO_2max_) is the best fitness evaluation tool, and it increases with pubertal development. However, obese children must exert themselves more in order to perform their daily activities; therefore, they reach their VO_2max_ earlier than eutrophics (percentile ≥ 25 and < 95) do. This may be related to anticipation of effort adaptive mechanisms, mechanisms that initiate earlier pubertal development in obese children. They have been described in the literature, but no consensus regarding the mechanisms has been reached [15–18].

Lower values of FEV_1_/FVC were observed in the obese group in the present study. There were no differences in FEV_1_ between the groups, and obese subjects demonstrated higher FVC values, but these values became statistically non-significant following Bonferroni correction. However, these results demonstrate that the lower FEV_1_/FVC values observed in the obese group were determined by higher FVC values as opposed to lower values of FEV_1_, which may be related to these patients’ increased need for oxygen due to greater oxygen consumption.

The literature diverges regarding spirometric findings in obese children and adolescents. There were no significant differences in FVC between the obese group and the healthy group in this study, whereas a study performed by another author found no relationship between body composition and FVC [19]. However, other studies noted higher values of FVC in obese children and adults [20–22], whereas additional studies noted lower FVC values in obese children [23, 24].

The same authors disagree regarding the relationship between FEV_1_ and obesity. The present study did not find differences in FEV_1_ between the groups, which was consistent with a finding in the literature [19]. Some studies noted lower values of FEV_1_ in the setting of obesity [23, 24], although others noted higher FEV_1_ values [20, 21].

Greater consistency has been noted in the literature regarding FEV_1_/FVC ratios. Other authors found that this variable was reduced in obese patients, a finding consistent with that of our study [20, 24, 25].

Another study observed a negative correlation between sagittal abdominal diameter and FEV_1_/FVC [22], which may be related to sex differences, as a similar finding was observed in the present study. Boys experience android obesity; therefore, fat distribution occurs primarily in abdominal and chest areas, and they develop larger sagittal abdominal diameters than girls, who experience gynoid fat distribution, which is characterized by the distribution of adipose tissue at the hips and in the thighs. Another study noted lower FEV_1_/FVC values only in obese boys, a finding that supports this hypothesis [26].

Forced expiratory flow reduction among obese subjects may be explained primarily by compromised lung mechanics as a result of the extra load that adipose tissue imposes upon the ribcage, a phenomenon supported by other literature articles [23, 25, 27]. However, a study involving 64 obese subjects with an average age of 12 years noted only three individuals with obstruction abnormalities [28], and another study that included 22 obese subjects between two and 20 years of age noted only one child with flow obstruction [29]. Low prevalences of obstruction disorders were also observed in other studies [19, 21].

In both the obese and healthy groups, it was observed that forced expiratory flow volumes were lower in males than in females, a finding that may be related to lung growth and structural differences between the sexes. Male lungs are larger than female lungs of the same age. Therefore, they have longer but narrower airways, which limits expiratory flow [30].

The 6MWT results are due to physiological changes described previously. The obese group walked a shorter distance than the control group due to extra load, weak musculature, sedentary lifestyle, and reduced glycolytic capacity. Nevertheless, these subjects did more work while walking, as they have higher cardiorespiratory requirements. There is a study in which a group of authors found that 6MWT performances were 26% worse in obese subjects between eight and 16 years old compared with eutrophics of same age. They concluded that the 6MWT is a reproducible test among obese children and adolescents and is useful in clinical practice, although it did not demonstrate a strong correlation with VO_2max_ [10].

Some studies suggest that poor performance on the 6MWT, as observed in the present study, persists into adulthood. They also found that obese individuals walk shorter distances than eutrophics and that obese subjects also perform more work [25, 31].

PC was originally proposed by McGregor [32] and may represent an alternative means of analyzing the 6MWT. The relationship between ∆HR and average speed estimates energetic expenditure, and studies have used this parameter to evaluate healthy subjects and individuals with diseases. Its applicability is still debated, however, because there are many variations among the studies that have utilized it [32–37]. No studies comparing PC in obese and healthy individuals have been found.

The obese group demonstrated lower PC values among subjects between five and 11 years of age, walked shorter distances and exhibited lower HR_6min_ than eutrophics, demonstrating lower values in ∆HR and average speed. With growth, lower values in HR and increases in walking speed are expected in healthy individuals due to increasing leg lengths and expected improvements in cardiorespiratory fitness. Therefore, obese subjects, who demonstrated reduced average speeds due to excessive load, demonstrate lower PC values upon reaching adulthood.

Analyzing normal parameters of the 6MWT has resulted in disagreements regarding the test’s criteria due to anthropometric variations in each ethnic group, age differences, and the importance of each variable in normality equation determination, each of which hampers the homogeneity of this particular evaluation.

Both Brazilian studies included similar samples based on subjects’ nationality, and also included subtractive body mass related variables accounting for body weight influences on performance. However, Li et al. [12] utilized criteria based on Chinese children and adolescents in their study, characteristics different from those of the Brazilian population, and observed that all obese subjects and almost all eutrophics performed below normal values. Therefore, these criteria were not suitable for the evaluations of obese and eutrophic differences in our sample. An equation developed by Geiger et al. [13] evaluated a population with different ethnic characteristics than those of Brazilians: they observed differences between the obese and healthy groups’ performances. However, the authors did not take into account the influence of body mass on performance when developing their normality equation [11–14].

### Limitations of the study

It is necessary to develop more accurate methods of defining obesity and to evaluate body composition more precisely. For example, researchers must define percentages of body fat and lean mass so that they will select better populations for study. Moreover, the control groups for spirometry and the 6MWT were composed of different individuals. Therefore, we could not establish a correlation among these variables in our healthy subjects. Finally, as sample numbers conform to sample calculations, a larger population may allow for the detection of significant differences among groups.

## Conclusion

Based on the results of the present study, the obese group demonstrated poor fitness, as evidenced by poor 6MWT performances. Spirometry changes related to forced expiratory flow reduction were also observed and were suggestive of obstructive abnormalities in 36.8% of our obese subjects. The obese group demonstrated lower FEV_1_/FVC values. Changes in lung function among our obsess subjects did not correlate directly with their performances on the 6MWT. However, there was a correlation between lung function and variables indicative of effort during exercise.

## Electronic supplementary material

Additional file 1: **Comparisons of obese subjects’ spirometry performances before and after bronchodilator use.** *p-value smaller than 0.05; the Wilcoxon test was used. (PDF 236 KB)

Additional file 2: **All clinical relationships between obesity and lung function markers.** (DOCX 14 KB)

Additional file 3: **All clinical relationships between obesity and lung function markers before and after bronchodilator treatments.** (DOCX 13 KB)

Additional file 4: **All clinical relationships between obesity and walking test markers.** (DOCX 14 KB)

Additional file 5: **All clinical relationships between obesity and walking test markers in obese patients.** (DOCX 13 KB)

## Abbreviations

∆HR: Pre- and post-walking heart rate difference
6’: Six minutes
6MWT: Six-minute walk test
9’: Nine minutes
ATS: American Thoracic Society
BD: Bronchodilator
BMI: Body mass index
BorgD: Dyspnea perception according to the Borg scale
BorgL: Legs effort perception according to the Borg scale
BP: Blood pressure
CDC: Center for Disease Control and Prevention
CI: Confidence interval
ERS: European Respiratory Society
ERV: Expiratory reserve volume
FEF_25%_: Forced expiratory flow at twenty five percent of the forced vital capacity
FEF_25–75%_: Forced expiratory flow between twenty five and seventy five percent of the forced vital capacity
FEF_50%_: Forced expiratory flow at fifty percent of the forced vital capacity
FEF_75%_: Forced expiratory flow at seventy five percent of the forced vital capacity
FEFmax: Maximum forced expiratory flow
FEV_1_/FVC: Tiffeneau index
FEV_1_: Forced expiratory volume at first second
FVC: Forced vital capacity
HR: Heart rate
N: Number of patients
OD: Odds ratio
p: P-value
PC: Physiological cost
p^c^: P-value corrected
RR: Respiratory rate
SpO_2_: Oxygen saturation
SPSS: Statistical Package for the Social Sciences
Unicamp: University of Campinas
VC: Vital capacity
VO_2max_: Maximal oxygen uptake
W: Work index
WD: Walking distance
WHO: World Health Organization
α: Alpha
χ^2^: Chi square.
