# Supplementary material for: The relationship between physical functional capacity and lung function in obese children and adolescents
Source: BMC Pulm Med. 2014 Dec 15;14:199. doi: 10.1186/1471-2466-14-199 (PMC4280742; doi:10.1186/1471-2466-14-199)

Supplementary 1. Comparisons of obese subjects' spirometry performances before and after bronchodilator use. *p-value smaller than 0.05; the Wilcoxon test was used.

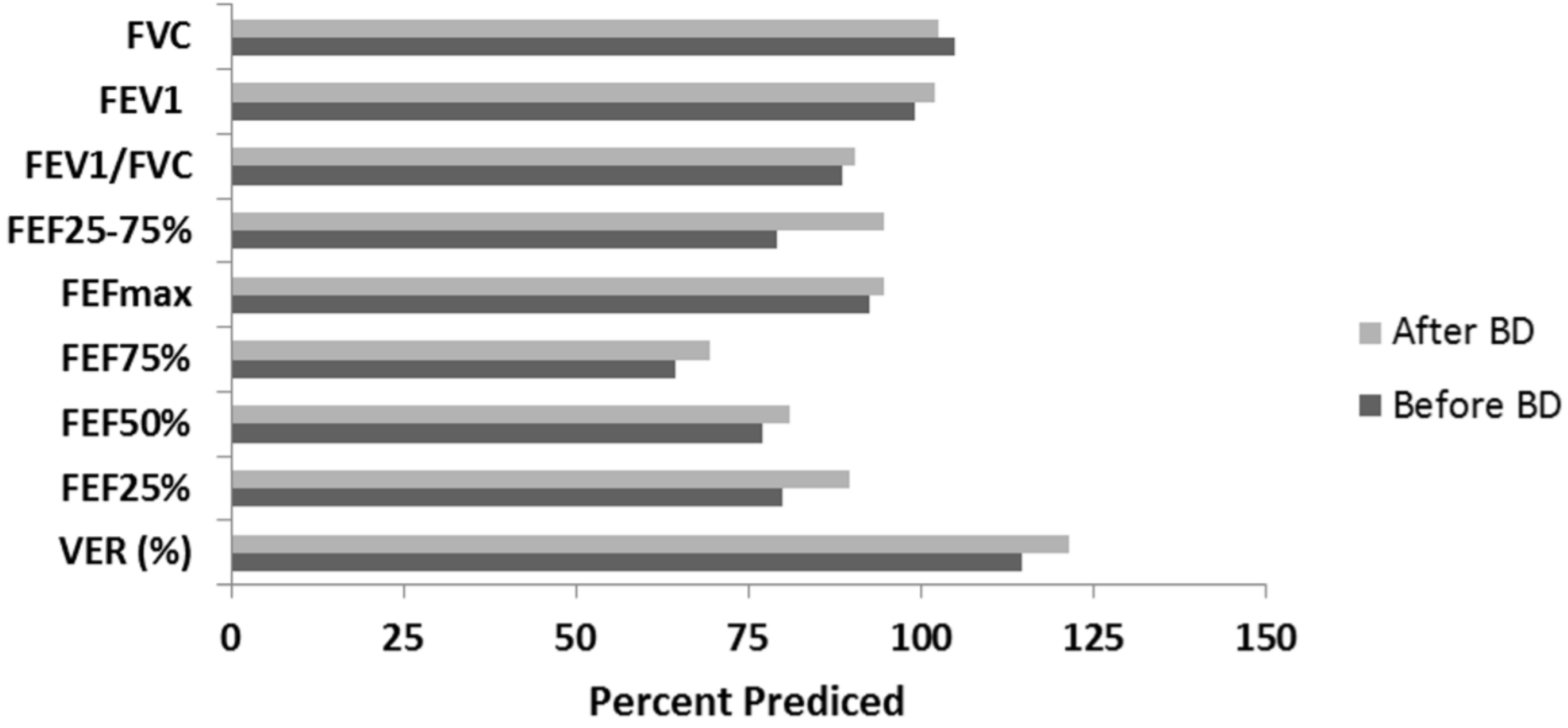

Supplement: Supplementary file 1 — Additional file 1: Comparisons of obese subjects’ spirometry performances before and after bronchodilator use. *p-value smaller than 0.05; the Wilcoxon test was used. (PDF 236 KB) [file 12890_2013_635_MOESM1_ESM.pdf]
