# Supplementary material for: The relationship between physical functional capacity and lung function in obese children and adolescents
Source: BMC Pulm Med. 2014 Dec 15;14:199. doi: 10.1186/1471-2466-14-199 (PMC4280742; doi:10.1186/1471-2466-14-199)
Supplement: Supplementary file 2 — Additional file 2: All clinical relationships between obesity and lung function markers. (DOCX 14 KB) [file 12890_2013_635_MOESM2_ESM.docx]

| **Supplementary 2.** All clinical relationships between obesity and lung function markers. | | | | | | |
| --- | --- | --- | --- | --- | --- | --- |
| Clinical marker | Obese versus eutrophic* | | Sex^#^ | | Age^#^ | |
|  | p | p^c^ | p | p^c^ | P | p^c^ |
| Weight | **<0.001** | **<0.003** | **<0.001** | **<0.003** | **<0.001** | **<0.003** |
| Height | 0.318 | 0.954 | 0.424 | 1 | **<0.001** | **<0.003** |
| Body mass index | **<0.001** | **<0.003** | **<0.001** | **<0.003** | **<0.001** | **<0.003** |
| FVC% | **0.028** | 0.084 | 0.099 | 0.297 | **0.017** | 0.051 |
| FEV_1%_ | 0.126 | 0.378 | 0.069 | 0.207 | 0.374 | 1 |
| FEV_1_/FVC | **<0.001** | **<0.003** | **<0.001** | **<0.003** | **<0.001** | **<0.003** |
| FEF_25%_ | **<0.001** | **<0.003** | **<0.001** | **<0.003** | **<0.001** | **<0.003** |
| FEF_50%_ | **<0.001** | **<0.003** | **<0.001** | **<0.003** | **<0.001** | **<0.003** |
| FEF_75%_ | **0.001** | **0.003** | **<0.001** | **<0.003** | **<0.001** | **<0.003** |
| FEF_25-75%_ | **<0.001** | **<0.003** | **<0.001** | **<0.003** | **<0.001** | **<0.003** |
| FEF maximum | **<0.001** | **<0.003** | **<0.001** | **<0.003** | **<0.001** | **<0.003** |
| Expiratory reserve volume | 0.630 | 1 | 0.169 | 0.507 | **0.009** | **0.027** |
| FEF_25-75%_ by class | **<0.001** | **<0.003** | **<0.001** | **<0.003** | **<0.001** | **<0.003** |

FEV_1_ = forced expiratory volume in 1 second; FVC = forced vital capacity; FEF = forced expiratory flow; p = p-value; p^c^ = p-value corrected using the Bonferroni test. *Statistical analyses were performed using the Mann-Whitney test, given an α = 0.05. ^#^ Statistical analyses were performed using the Kruskal–Wallis one-way analysis of variance test, given an α = 0.05. Positive p-values are shown in bold.
