# Supplementary material for: The relationship between physical functional capacity and lung function in obese children and adolescents
Source: BMC Pulm Med. 2014 Dec 15;14:199. doi: 10.1186/1471-2466-14-199 (PMC4280742; doi:10.1186/1471-2466-14-199)
Supplement: Supplementary file 3 — Additional file 3: All clinical relationships between obesity and lung function markers before and after bronchodilator treatments. (DOCX 13 KB) [file 12890_2013_635_MOESM3_ESM.docx]

| **Supplementary 4.** All clinical relationships between obesity and lung function markers before and after bronchodilator treatments. | | | | |
| --- | --- | --- | --- | --- |
| Clinical marker | Sex | | Age | |
|  | p | p^c^ | p | p^c^ |
| FVC% | 0.663 | 1 | 0.794 | 1 |
| FEV_1%_ | 0.728 | 1 | 0.706 | 1 |
| FEV_1_/FVC | 0.685 | 1 | 0.685 | 1 |
| FEF_25%_ | 0.322 | 0.644 | 0.467 | 0.934 |
| FEF_50%_ | 0.663 | 1 | 0.542 | 1 |
| FEF_75%_ | 0.581 | 1 | 0.750 | 1 |
| FEF_25-75%_ | 0.642 | 1 | 0.908 | 1 |
| FEF maximum | 0.416 | 0.832 | 0.839 | 1 |
| Expiratory reserve volume | 0.977 | 1 | 0.772 | 1 |

FEV_1_ = forced expiratory volume in 1 second; FVC = forced vital capacity; FEF = forced expiratory flow; p = p-value; p^c^ = p-value corrected using the Bonferroni test. *Statistical analyses were performed using the Mann-Whitney test, given an α = 0.05. Positive p-values are shown in bold.
