# Supplementary material for: The relationship between physical functional capacity and lung function in obese children and adolescents
Source: BMC Pulm Med. 2014 Dec 15;14:199. doi: 10.1186/1471-2466-14-199 (PMC4280742; doi:10.1186/1471-2466-14-199)
Supplement: Supplementary file 4 — Additional file 4: All clinical relationships between obesity and walking test markers. (DOCX 14 KB) [file 12890_2013_635_MOESM4_ESM.docx]

| **Supplementary 4.** All clinical relationships between obesity and walking test markers. | | | | | | |
| --- | --- | --- | --- | --- | --- | --- |
| Clinical markers | Obese versus eutrophic* | | Sex^#^ | | Age^#^ | |
|  | p | p^c^ | p | p^c^ | p | p^c^ |
| Walking distance | **<0.001** | **<0.003** | **<0.001** | **<0.003** | **<0.001** | **<0.003** |
| Cardiac frequency - rest | 0.200 | 0.600 | 0.518 | 1 | **0.025** | 0.075 |
| Respiratory frequency - rest | **0.045** | 0.135 | **0.031** | 0.093 | 0.069 | 0.207 |
| Peripheral oxygen saturation - rest | 0.712 | 1 | 0.177 | 0.531 | **0.041** | 0.123 |
| Borg – rest | 0.119 | 0.238 | 0.451 | 1 | 0.054 | 0.162 |
| Cardiac frequency - six minutes | **0.001** | **0.003** | **0.004** | **0.012** | **0.007** | **0.021** |
| Respiratory frequency - six minutes | **0.012** | **0.036** | **0.047** | 0.141 | **0.015** | **0.045** |
| Peripheral oxygen saturation - six minutes | 0.249 | 0.747 | 0.672 | 1 | 0.145 | 0.435 |
| Borg - six minutes | 0.266 | 0.798 | 0.473 | 1 | 0.299 | 0.897 |
| Cardiac frequency - nine minutes | **0.014** | **0.042** | **0.050** | 0.15 | **0.012** | **0.036** |
| Respiratory frequency - nine minutes | **<0.001** | **<0.003** | **<0.001** | **<0.003** | **<0.001** | **<0.003** |
| Peripheral oxygen saturation - nine minutes | 0.053 | 0.159 | 0.233 | 0.699 | **0.049** | 0.147 |
| Borg - nine minutes | **0.024** | 0.072 | 0.142 | 0.426 | 0.155 | 0.465 |
| Walking distance x weight | **<0.001** | **<0.003** | **<0.001** | **<0.003** | **<0.001** | **<0.003** |
| Physiology cost index | 0.853 | 1 | 0.805 | 1 | 0.007 | 0.021 |
| Li | **0.025** | 0.075 |  |  |  |  |
| Geiger | **<0.001** | **<0.003** |  |  |  |  |
| Oliveira | **<0.001** | **<0.003** |  |  |  |  |
| Priesnitz | **<0.001** | **<0.003** |  |  |  |  |

p = p-value; p^c^ = p-value corrected by Bonferroni test. *Statistical analyses were performed using the Mann-Whitney test, given an α = 0.05. ^#^ Statistical analyses were performed using the Kruskal-Wallis one-way analysis of variance test, given an α = 0.05. Positive p-values are shown in bold.
