# Supplementary material for: The relationship between physical functional capacity and lung function in obese children and adolescents
Source: BMC Pulm Med. 2014 Dec 15;14:199. doi: 10.1186/1471-2466-14-199 (PMC4280742; doi:10.1186/1471-2466-14-199)
Supplement: Supplementary file 5 — Additional file 5: All clinical relationships between obesity and walking test markers in obese patients. (DOCX 13 KB) [file 12890_2013_635_MOESM5_ESM.docx]

| **Supplementary 5.** All clinical relationships between obesity and walking test markers in obese patients. | | | | |
| --- | --- | --- | --- | --- |
| Clinical markers | Sex | | Age | |
|  | p | p^c^ | P | p^c^ |
| Systolic blood pressure - rest | 0.249 | 0.498 | 0.068 | 0.136 |
| Diastolic blood pressure - rest | 0.449 | 0.898 | **0.050** | 0.100 |
| Perceived exertion in lower limbs by BORG - rest | 0.531 | 1 | 0.812 | 1 |
| Systolic blood pressure - six minutes | **0.036** | 0.072 | **0.028** | 0.056 |
| Diastolic blood pressure - six minutes | 0.308 | 0.616 | 0.050 | 0.100 |
| Perceived exertion in lower limbs by BORG - six minutes | 0.746 | 1 | 0.503 | 1 |
| Systolic blood pressure - nine minutes | **0.025** | 0.050 | **0.036** | 0.072 |
| Diastolic blood pressure - nine minutes | 0.531 | 1 | 0.092 | 0.184 |
| Perceived exertion in lower limbs by BORG - nine minutes | 0.215 | 0.430 | 0.398 | 0.796 |

p = p-value; p^c^ = p-value corrected by Bonferroni test. Statistical analyses were performed using the Mann-Whitney test, given a α = 0.05. Positive p-values are shown in bold.
